# From genetic variants to therapeutic targets: insights into understanding rheumatoid arthritis

**DOI:** 10.3389/fimmu.2025.1556971

**Published:** 2025-04-01

**Authors:** Lu Chen, Jianan Zhao, Qingliang Meng

**Affiliations:** ^1^ Department of Traditional Chinese Medicine, Aviation General Hospital, Beijing, China; ^2^ Department of Rheumatology, Guanghua Hospital Affiliated to Shanghai University of Traditional Chinese Medicine, Shanghai University of Traditional Chinese Medicine, Shanghai, China; ^3^ Department of Rheumatism, Henan Province Hospital of Traditional Chinese Medicine, Zhengzhou, Henan, China

**Keywords:** rheumatoid arthritis, genetic susceptibility, precision medicine, novel mechanism, inflammation

## Abstract

Rheumatoid arthritis (RA) is a chronic inflammatory autoimmune disease that affects multiple systems and is driven by various factors, including interactions between genetic and environmental elements. Over the past few decades, genome-wide association studies (GWAS) have been instrumental in regard to identifying genetic and environmental risk factors associated with RA susceptibility and pathogenesis. The recent discoveries of novel genetic susceptibility loci and pathways offer promising therapeutic targets for RA and precision medicine. More than 100 genetic loci have been identified in RA patients. In this review, we have focused on more than 40 genes that have been supported by evidence to be closely associated with the development of RA. These include genes involved in various mechanisms, such as loss of self-tolerance, autoimmune antibody production (e.g., *HLA-DRB1, HLA-DPB1*), inflammatory signaling and bone destruction (e.g., *PTPN22, CCR6*), complication (e.g., *HLA-DQB1, IRF5*), and differential drug responses (e.g., *HLA-E, NKG2D*). These novel players and mechanisms enhance our understanding of the RA pathogenesis and also provide a reference for personalized and precise medicine, including diagnosis and treatment.

## Introduction

Rheumatoid arthritis (RA) is a chronic systemic autoimmune disease characterized by persistent synovial inflammation, synovial hyperplasia, and progressive destruction of bone and cartilage. These pathological processes can lead to joint deformities and loss of function, significantly affecting patients’ quality of life. In addition to joint damage, RA often manifests with extraarticular complications involving the cardiovascular, pulmonary, and other organ systems. These systemic effects impose a substantial burden on patients’ physical and mental well-being, as well as their socioeconomic status (1). Despite their complexity, advances in therapeutic approaches have remarkably improved disease outcomes over the past few decades. Targeted immune-modulating therapies have emerged as effective strategies to mitigate disease progression and shift the clinical management landscape of RA.

However, RA is a highly heterogeneous disease with substantial variability in disease onset, progression, and therapeutic responses. This variability is influenced by the interplay of genetic, molecular, and environmental factors, leading to distinct cellular responses, molecular profiles, and signaling pathway alterations across individuals (2–5). Such heterogeneity poses challenges to achieving consistently effective treatments and underscores the importance of precision medicine. Notably, while certain RA therapies are highly effective for some patients, others experience limited or no response, thus highlighting the critical need to refine our understanding of the molecular underpinnings of the disease to guide personalized treatment strategies.

In recent years, genome-wide association studies (GWAS) and other high-throughput genetic analyses have revolutionized our understanding of RA pathogenesis. These advanced techniques have facilitated the identification of numerous genetic factors and loci associated with the susceptibility, progression, and treatment response of RA. For instance, a comprehensive analysis published in 2018 updated the list of RA-associated loci to more than 106, shedding light on the intricate genetic architecture underlying this disease (6). Among these, the human leukocyte antigen (HLA) region is a major contributor to RA susceptibility, specifically HLA-DRB1 shared epitope alleles. In addition to HLA, non-HLA genes such as peptidyl arginine deiminase 4 (*PADI4)*, protein tyrosine phosphatase, non-receptor type 22 (*PTPN22)*, and signal transducer and activator of transcription 4 (*STAT4*) have also been implicated in RA pathogenesis, further underscoring the genetic complexity of the disease. Given the rapid advancements in the identification of RA-related genes, understanding how these genetic factors influence the pathological mechanisms is crucial. These mechanisms include the breakdown of self-tolerance, autoimmune antibody production, dysregulated inflammatory signaling, tissue destruction, and variability in therapeutic efficacy. Therefore, we conducted a focused review to explore genes supported by strong evidence linking them to RA. Specifically, we have aimed to identify genes involved in critical pathways, such as autotolerance loss, autoantibody formation, inflammatory signaling, and joint destruction. Moreover, we have examined the genetic factors that may contribute to variable responses to treatment. By synthesizing the findings from the latest literature, this review provides a framework for understanding the genetic basis of RA and also its implications for personalized and precision medicine.

## Gene variants contribute to the breaking of autoimmune tolerance status and enhance the susceptibility to RA

Loss of self-tolerance in RA is a critical event that leads to the erroneous targeting of host tissues by the immune system. Genetic susceptibility, in concert with environmental factors, predisposes individuals to this breakdown in which autoantigens elicit immune responses. Central to this process is the HLA complex, which encodes proteins integral to the major histocompatibility complex (MHC). The HLA complex is one of the most polymorphic regions in the human genome and its diversity plays a pivotal role in antigen processing and presentation.

The HLA region is divided into class I and class II genes (7). Class I genes, such as major histocompatibility complex, class I, A (*HLA-A*), Class I genes, such as major histocompatibility complex, class I, B (*HLA-B)*, and Class I genes, such as major histocompatibility complex, class I, C (*HLA-C*) primarily present endogenous antigens against CD8+ T cells, whereas class II genes, including major histocompatibility complex, class II, DR beta 1(*HLA-DRB1*), major histocompatibility complex, class II, DQ beta 1(*HLA-DQB1*), and major histocompatibility complex, class II, DP beta 1(*HLA-DPB1*) present exogenous antigens against CD4+ T helper cells. Notably, the *HLA-DRB1*-shared epitope alleles (e.g., *04:01, *04:04, and DR4/DRx heterozygotes) demonstrate strong RA association through three principal mechanisms: (1) shaping thymic selection by altering T-cell receptor (TCR) affinity thresholds, permitting escape of autoreactive clones (8); (2) facilitating post-translational citrullination of bound peptides through local microenvironment modifications (9); and (3) enabling cross-reactivity between microbial and self-peptides via conserved binding grooves (9).

Beyond *HLA*, functional polymorphisms in immune regulatory genes amplify RA risk through distinct pathways. The *IL23R* and *PTPN22* are the two most relevant non-HLA factors associated with RA (10). The *PTPN22* C1858T variant (rs2476601) disrupts lymphoid tyrosine phosphatase (LYP) function, attenuating TCR signaling thresholds and promoting autoreactive lymphocyte survival through impaired negative selection (11). Similarly, the *IL23R* rs11209026 (Arg381Gln) variant modulates Th17 differentiation by altering IL-23 receptor signaling, potentially enhancing synovial inflammation through IL-17A overproduction (12). These non-HLA variants collectively illustrate how genetic perturbations across multiple immunological checkpoints—from antigen presentation (HLA) to lymphocyte activation (*PTPN22*) and effector cell differentiation (*IL23R*)—synergistically breach tolerance mechanisms.

## Effect of gene variants on autoimmune antibodies in RA

Autoimmune antibodies in RA, particularly rheumatoid factor (RF) and anti-cyclic citrullinated peptide antibodies (ACPA), are commonly detected before the clinical onset of the disease. These antibodies serve as important biomarkers of disease progression and prognosis (13). In particular, RF is considered a strong predictor of more severe and destructive forms of RA (14). Genetic variants have been shown to influence the production of autoimmune antibodies, thereby contributing to the pathogenesis and clinical manifestations of RA. For example, in Greek patients with RA, the homozygosity of the MHC class I polypeptide-related sequence A (*MICA*) rs1051792-GG allele was found to be associated with elevated levels of ACPA and RF, indicating a genetic predisposition to antibody production that might influence disease severity. Mechanistically, *MICA* encodes a stress-induced protein recognized by natural killer (NK) cells and γδ T cells. The rs1051792-GG variant may enhance *MICA*-mediated activation of these immune cells, promoting B cell differentiation and autoantibody production (15).

An important aspect of RA genetics is the different genetic backgrounds of ACPA-positive and ACPA-negative RA patients. Gene variants are among the most important contributors in regard to antibody production in ACPA-positive RA patients. One such example is the single-nucleotide polymorphism (SNP) kinesin family member 5A(*KIF5A*)*-* phosphatidylinositol 5-phosphate 4-kinase type 2 gamma *(PIP4K2C*)/rs1678542, which has been found to be associated with significant genetic susceptibility in the Pakistani RA population. This SNP is involved in immune signaling through B cell antigen receptors, which play a key role in the generation of autoimmune antibodies (16). These antibodies, produced by activated B cells, are crucial in the early stages of RA and correlate with the severity of joint damage over time. Additionally, *HLA-DPB1* is a susceptibility factor for ACPA-positive RA. In particular, HLA-DPB1*04:02 and HLA-DPB1*02:01 have been shown to be significantly associated with ACPA positivity in the Japanese population, while HLA-DPB1*05:01 seems to reduce the risk of ACPA-positive RA (17). HLA-DPB1*04:02 and HLA-DPB1*02:01 alleles encode peptide-binding grooves with high affinity for citrullinated antigens. This structural feature facilitates the presentation of modified self-peptides to CD4 + T cells, driving autoimmune B cell responses and ACPA production. Conversely, HLA-DPB1*05:01 may lack this binding capacity, thereby reducing ACPA risk.

Moreover, genetic variations in *HLA-DPB1*, specifically SNP rs9277535, have been found to modulate genetic susceptibility to RA in the Chinese Han population. The presence of AG and GG + AG genotypes was linked to higher levels of C-reactive protein (CRP), ACPA, and erythrocyte sedimentation rate (ESR), compared to the AA genotype (18). This SNP alters *HLA-DPB1* expression, potentially increasing antigen presentation efficiency and amplifying T cell-dependent autoantibody production. Furthermore, the *A* allele of *HLA-DPB1* is associated with elevated serum ACPA levels, likely by enhancing citrullinated peptide presentation to autoreactive T cells (19).

Beyond HLA genes, the methylenetetrahydrofolate reductase (*MTHFR*) *1298CC* genotype may influence ACPA titers by disrupting folate metabolism. Impaired folate cycling can lead to hyperhomocysteinemia, increasing oxidative stress and post-translational protein modifications (e.g., citrullination), which in turn generate neo-antigens that trigger ACPA production (20, 21). In addition, environmental factors, particularly smoking, have also been implicated in the development of ACPA, with genetic variants at loci such as butyrophilin like 2 (*BTNL2)*, Major histocompatibility complex, class II, DR alpha (*HLA-DRA*), *HLA-DRB5*, major histocompatibility complex, class II, DQ Alpha 1(*HLA-DQA1*), major histocompatibility complex, class II, DO Beta (*HLA-DOB*), and transporter 2, ATP-binding cassette subfamily b member(*TAP2*) interacting with smoking to increase the likelihood of developing ACPA-positive RA (22). In summary, these studies emphasized the relationship between genetic variation and ACPA production.

Genetic research has indicated that ACPA+ RA and ACPA– RA are distinct diseases with varying underlying pathophysiology (23). In patients with ACPA-negative RA, certain polymorphisms have been identified, including variants in the ankyrin repeat domain 55(*ANKRD55*)/interleukin 6 signal transduce (*IL6ST*), B lymphoid tyrosine kinase (*BLK*), tumor necrosis factor alpha-induced protein 3 (*TNFAIP3*), GIN1 protein/C5orf30 (*GIN1/C5orf30*)*, STAT4*, 60S ribosomal protein pseudogene (*RPL*), and nuclear factor I A (*NFIA*)genes, suggesting that distinct genetic factors may underlie these two subsets of RA (24, 25). In multiethnic populations such as Malaysia, HLA-DQB1*03:02 was found to be negatively correlated with ACPA positivity, further emphasizing the role of specific HLA gene variants in modulating autoantibody production (26). Other studies have shown that certain inflammatory markers, such as C-C motif chemokine ligand (CCL)13, CCL18, and MMP3 are upregulated in ACPA-negative patients with RA, suggesting a distinct inflammatory profile in this subset of the disease (27). In contrast, *HLA-DRB5* expression was absent in ACPA-negative patients, whereas cytotoxic gene expression was decreased, providing further evidence for the complex immune interactions involved in RA (22, 28, 29).

Some studies have highlighted the genetic advancements in RF in patients with RA. For example, the HLA-DQB1*03/DQA1*03 and HLA-DRB1*04 alleles are associated with a higher RF prevalence, whereas HLA-DQB1*0501/DQA1*01 and HLA-DRB1*10 alleles seem to confer a protective effect against RA by reducing RF positivity (30). Additionally, RA patients carrying the rs2073618 osteoprotegerin variant (*OPG*) exhibit higher RF antibody levels and increased disease activity. The *OPG* rs2073618 variant may also exacerbate RA severity by disrupting the RANKL/OPG axis, which typically restricts osteoclast activation and may influence B cell survival in inflamed joints (20). The presence of the *(70)DERAA(74)* motif of *HLA-DQB1* in certain patients with RA also appears to decrease the risk of developing rheumatoid nodules and is associated with lower RF seropositivity. This protective effect may arise from the motif’s reduced binding affinity to citrullinated self-peptides, thereby limiting aberrant CD4+T cell activation and subsequent B cell-driven RF production (30). In Danish patients with RF-negative RA, there was a significant association between the Toll-like receptor (*TLR3*) rs3775291 *A* allele and disease activity (31). Despite these insights, several past studies have reported contradictory findings, likely due to the heterogeneity of RA, including variations in regional populations, sex, and other environmental factors. These inconsistencies highlight the need for more focused studies that consider these diverse factors to better stratify patients with RA and refine therapeutic approaches.

## Effect of gene variants on inflammation and bone destruction in RA

RA involves complex interactions between immune cells, and genetic variants significantly influence inflammatory processes and subsequent bone destruction. HLA class II molecules such as *HLA-DRB1* are pivotal for presenting antigens to CD4+ T cells, leading to the activation of autoreactive T cells such as Th1 and Th17 cells. Once activated, these T cells stimulate inflammatory responses by releasing pro-inflammatory cytokines such as interleukin-1 (IL-1), interleukin-6 (IL-6), and tumor necrosis factor-alpha (TNF-α), contributing to synovial inflammation and joint destruction.

Variations in HLA class II molecules have been associated with changes in inflammatory markers, including CRP and ESR, which are used to assess disease activity in patients (See **Table 1**). In female patients with RA, certain *HLA-DRB1* variants, namely *03, *04, *08, *10, *11, and * 14, were significantly correlated with an elevated ESR, indicating increased inflammation. Conversely, HLA-DRB1*03, *13, and *15 negatively correlated with elevated CRP levels, suggesting a potential protective effect against systemic inflammation (28). Non-class II HLA genetic variants also play a significant role in RA pathogenesis. The *HLA-DQA*1 160D variant in the Chinese Han population is associated with ACPA positivity and is believed to stabilize dimers, enhance T-cell activation, and contribute to inflammation (35). In contrast, HLA-DQA1*3031 appears to protect against RA, whereas HLA-DQA1*0302 and *0303 interact with *HLA-DR4* to increase the risk of bone erosion and RF seropositivity, emphasizing their impact on bone health (36).

**Table 1:** Gene variants linked to RA.

	Gene	Variants	Effect	Ref.
Autoantibodies	*MICA*	rs1051792	It associated with elevated levels of rheumatoid factor (RF) and anti-cyclic citrullinated peptide antibodies (ACPA).	(15, 32, 33)
	*KIF5A*	rs1678542	It participates in autoimmune signal transmission through B cell antigen receptors.	(16, 34)
	*HLA-DPB1*	HLA-DPB1*04:02, HLA-DPB1*02:01, HLA-DPB1*05:01, rs9277535,	It associated with ACPA.	(17, 18)
	*MTHFR*	1298CC	It associated with ACPA.	(20)
	*HLA-DQB1*	HLA-DQB1*03:02, HLA-DQB1*03, HLA-DQB1*0501	It is correlated with ACPA and RF.	(26)
	*HLA-DRB5*	HLA-DRB5*01	It is associated with ACPA –positive RA and serves as a protective factor for RA.	(22, 28, 29)
	*HLA-DQA1*	HLA-DQA1*03, HLA-DQA1*01	It is associated with RF.	(30)
	*HLA-DRB1*	HLA-DRB1*04, HLA-DRB1*10	It is associated with RF.	(30)
	*OPG*	rs2073618	It is associated with RF.	(20)
	*TLR3*	rs3775291	It is associated with RF.	(31)
Inflammation and joint erosion	*HLA-DRB1*	HLA-DRB1*03, HLA-DRB1*04, HLA-DRB1*08, HLA-DRB1*10, HLA-DRB1*11, HLA-DRB1*14, HLA-DRB1*13, HLA-DRB1*15	It is related to CRP, and ESR.	(28)
	*HLA-DQA1*	*HLA-DQA*1 160D, HLA-DQA1*3031, HLA-DQA1*0302, *HLA-DQA1* *0303,	It is related to T cell activation, inflammation, and bone destruction.	(35, 36)
	*MECP2*	rs17435	It regulates abnormal methylation patterns and SFRP4/Wnt pathway in RA and participates in the inflammatory response.	(37–40)
	*PTPN22*	*PTPN22* 620W	It dephosphorylates Src family kinases and negatively regulates TCR signaling.	(41)
	*CCR6*	rs1854853	It is related to T cell response.	(42, 43).
	*DPP4*	rs12617656	It is related to T cell response.	(42, 43).
	*STAT4*	rs11889341, rs7574865, rs8179673, rs10181656	It is related to T-cell receptor signaling or activation pathways.	(44)
	*TRAF1/C5*	rs10818488, rs3761847, rs2900180	It is related to T-cell receptor signaling or activation pathways.	(44)
	*Chr6q23*	rs6920220, rs13207033	It is related to T-cell receptor signaling or activation pathways.	(44)
	*KIF5A*	rs1678542	It is related to T-cell receptor signaling or activation pathways.	(44)
	*PRKCQ*	rs4750316	It is related to T-cell receptor signaling or activation pathways.	(44)
	*CD40*	rs4810485	It is associated with joint destruction in patients with ACPA-positive RA	(32)
	*CDK6*	rs42041	It is associated with joint destruction in patients with ACPA-positive RA	(32)
	*FCRL3*	*FCRL3* CC	It contribute to the progression of bone destruction in patients with RA	(45)
RA complication	*IRF5*	rs2004640, rs4728142, rs2004640, rs10954213	It is related to RA complicated by cardiovascular events and is involved in chronic inflammation and bone erosion.	(46, 47)
	*HLA-DQB1*	HLA-DQB1*03:01, HLA-DQB1*03:02	It is related to RA complicated with lung disease and chronic iridocyclitis.	(48)
	*HLA-DRB1*	HLA-DRB1*1104, HLA-DRB1*0201, HLA-DRB1*04, HLA-DRB1*0401, HLA-DRB1*1402, HLA-DRB1*0101, HLA-DRB5*01	It is related to RA complicated with chronic iridocyclitis and vasculitis.	(29, 49–51)
	*HLA-DQA1*	HLA-DQA1*0501, HLA-DQA1*0101	It is related to RA complicated with chronic iridocyclitis.	(49)
	*HLA-C*	HLA-C*0802	It serves as a significant predictive factor for vasculitis	(52)
Drug efficacy	*NKG2D*	rs2255336, rs1049174	It can predict the efficacy of anti-TNF-a therapy and related to inflammation.	(53)
	*HLA-DQB1*	HLA-DQB1*05	It is related to anti-TNF-a therapy.	(54)
	*HLA-DRB1*	HLA-DRB1*01, HLA-DRB1*07, HLA-DRB1*03, HLA-DRB1*011,	It is related to anti-TNF-a therapy.	(54)
	*HLA-DQA1*	HLA-DQA1*05	It is related to anti-TNF-a therapy.	(55)
	*HLA-E*	HLA-E*01:01/01:01, HLA-E*01:03	It is related to anti-TNF-a therapy.	(56)
	*MICA*	rs1051792	It can predict the efficacy of anti-TNF-a therapy.	(33, 57)
	*PDZD2*	rs1532269	It is related to anti-TNF-a therapy.	(58)
	*EYA4*	rs17301249	It is related to anti-TNF-a therapy.	(58)
	*IL17A*	*IL17A-152* G/A, *IL17F 7383* A/G, *IL17F 7488* A/G	It is related to methotrexate treatment.	(59)
	*NLRP3*	rs4925648, rs4925659, rs10925026	It is related to anti-TNF-a therapy.	(60)

In addition to HLA gene variants, non-HLA genetic variations also contribute to RA inflammation and bone destruction (see **Table 1**). For inflammation, the methyl-CpG-binding protein 2 (*MECP2*) SNP (rs17435) enhances RA susceptibility by affecting pathways that regulate inflammatory gene expression, such as the SFRP4 and Wnt pathways, and DNA methylation patterns (37–40). Another widely studied gene, *PTPN22*, encodes a protein phosphatase that affects T and B cell signaling (61, 62). It mainly encodes a tyrosine phosphatase that can negatively regulate T-cell receptor signal transmission by dephosphorylating members of the Src family of kinases. Carriers of the *620W* allele exhibited reduced T cell receptor (TCR) and B cell receptor (BCR) signals, which affected the clonal selection of lymphocytes and led to the emergence of autoimmune cells (63). Moreover, variants of *the 620W* allele are associated with the hyper-citrullinated state of peripheral blood mononuclear cells and NETosis formation, both of which increase the risk of RA inflammation (64). The *620W* allele also promotes disease progression by changing the cytokine profile of patients with RA, including profiles of interleukin (IL)-4, IL-10, interferon (IFN)-r, and TNF-α (41). One significant mediator of inflammation is interleukin-17 (IL-17), produced by Th17 cells, and genetic variants, such as C-C motif chemokine receptor 6 *(CCR6)* gene, a chemokine receptor critical for Th17 cell migration to inflamed joints, modulate IL-17 levels. Gene-gene interactions between *CCR6* SNP (rs1854853) and *DPP4* SNP (rs12617656) have been implicated in T cell responses in RA, thereby enhancing disease susceptibility (42, 43). Furthermore, poly(ADP-ribose) polymerase 1 (PARP-1) regulates CCR6 expression by modulating chromatin accessibility at the *CCR6* promoter through its enzymatic activity. PARP-1-mediated ADP-ribosylation of histones facilitates transcriptional CCR6 activation. This genetic control over inflammatory gene expression may exacerbate synovial inflammation in RA (65). Despite these insights, a large meta-analysis suggested the necessity for larger studies to address CCR6’s role, highlighting it as a potential therapeutic target (66). Other loci, including *STAT4*, tnf receptor associated factor 1(*TRAF1*)*/C5*, Chr6q23, kinesin family member 5A (*KIF5A*), and protein kinase C theta (*PRKCQ*)are involved in juvenile idiopathic arthritis (44). Continuous inflammation can further lead to bone destruction in RA, and research has shown that the Fc receptor-like 3(*FCRL3*)CC genotype is associated with an elevated risk of RA. This polymorphism alters the binding affinity of nuclear factor κB (NF-κB) and modulates *FCRL3* expression. *FCRL3* upregulation may enhance B-cell receptor signaling, thereby increasing the RANKL production. This process promotes osteoclasts activation and subsequent bone resorption (45, 67). Polymorphisms rs4810485 in CD40 molecule(*CD40*)and rs42041 in cyclin-dependent kinase 6(*CDK6*)are associated with a higher rate of joint destruction in patients with ACPA-positive RA (32). The *CD40* variant may enhance CD40–CD40 ligand (CD40L) interactions, thereby amplifying TNF-α and IL-6 production through T cell-dependent B cell responses and efficient T cell priming (68). CDK6 is a key protein involved in cell cycle regulation, particularly during the G1 phase transition. It associates with D-type cyclins and promotes cell cycle progression by pRb protein inactivation (69). *CDK6* polymorphisms may influence cell proliferation, such as that of synovial fibroblasts, by affecting CDK6 function. This dysregulation of cell cycle progression could exacerbate the pathological processes underlying RA.

## Effect of gene variants on complication for RA

Gene variants significantly contribute to the diversity of complications that have been observed in RA, affecting the cardiovascular, pulmonary, and ocular systems, among other extra-articular manifestations. For instance, specific SNPs in the interferon regulatory factor 5(*IRF5*)gene, including rs2004640, rs4728142, and rs10954213, have been linked with an increased risk of cardiovascular events in RA patients. IRF5 is a regulator induced by type I interferons and is closely associated with the production of inflammatory cytokines. *IRF5* variations may influence the production of these inflammatory cytokines, thereby playing a role in atherosclerosis development (46, 47). This emphasizes the need for the targeted monitoring of cardiovascular health in patients harboring these genetic markers to mitigate potential heart-related issues.

In terms of pulmonary complications, variations in the *HLA-DQB1* alleles have contrasting effects on patients with RA. HLA-DQB1*03:01 is associated with a higher risk of developing pulmonary diseases, including interstitial lung disease, which is a common and often severe complication of RA. Conversely, HLA-DQB1*03:02 served as a protective allele. HLA-DQB1*03:01 may facilitate the presentation of lung-specific self-antigens to autoreactive T cells, driving inflammation and fibrosis. Those suggest potential genetic pathways can be targeted to prevent lung involvement in RA (48). Recognizing these genetic predispositions can assist clinicians in regard to stratifying patients according to their risk of developing respiratory complications, thereby enabling personalized preventative strategies and interventions.

Ocular complications such as chronic iridocyclitis are a significant concern in pauciarticular juvenile RA. The risk of developing this condition increases in patients with specific HLA alleles, including HLA-DRB1*1104, HLA-DQA1*0501, HLA-DQB1*0301, and HLA-DRB1*0201 *(* 49). However, the presence of the HLA-DQA1*0101 allele is associated with reduced risk, indicating its potential protective role (70). These insights into genetic susceptibility underscore the importance of early eye examinations and proactive management of those at higher risk.

Additionally, certain genetic variants provide protective effects against systemic complications of RA. Rheumatoid vasculitis is an important extra-articular manifestation of RA that can lead to deep skin ulcers. Genetic variations in *HLA-DRB* appear to be associated with rheumatoid vasculitis, particularly the presence of two HLA-DRB1*04 alleles that encode a shared epitope (SE) linked to this condition (50). The SE motif facilitates the presentation of citrullinated peptides to autoreactive T cells, driving vascular inflammation and endothelial damage. Furthermore, HLA-DRB1*0401 has been associated with Felty syndrome. This association may arise from the allele’s ability to promote high-affinity T cell responses against self-antigens in hematopoietic tissues (50). A study on RA-related vasculitis in Brazil identified that HLA-DRB1*1402 and HLA-DRB1*0101 alleles may offer protection against skin lesions in affected individuals (51). These alleles may encode peptide-binding grooves with lower affinity for citrullinated antigens, thereby attenuating T cell-mediated vascular inflammation. Similarly, the increased frequency of the HLA-DRB5*01 allele in patients without vasculitis suggests its protective role against severe manifestations. HLA-DRB5*01 may modulate immune pathways by promoting regulatory T cell responses or limiting pro-inflammatory Th17 cell activation, thereby mitigating excessive inflammation and tissue damage (29). In addition to *HLA-DRB*, research has identified a positive correlation between the *HLA-C3* allele and vasculitis, indicating that *HLA-C3* serves as a significant predictive factor for this condition. The interaction of *HLA-C* products with the T-cell receptor on CD8+ T cells, as well as with killer cell immunoglobulin-like receptor molecules on NK cells and a certain T cell subsets, may influence RA-associated vasculitis development (52).

## Effect of gene variants on drug efficacy for RA

The therapeutic response to RA treatment varies considerably among patients, reflecting the intricate interplay between genetic predisposition and drug efficacy. Numerous gene variants have been identified that influence the effectiveness of anti-tumor necrosis factor (TNF-α) therapy and other biologics, providing insights into personalized treatment pathways and strategies to optimize clinical outcomes.

RA patients with specific HLA alleles demonstrate varied responses to adalimumab, a widely used anti-TNF-α therapy. For example, carriers of HLA-DQB1*05, HLA-DRB1*01, and HLA-DRB1*07 have been observed to experience better therapeutic outcomes with adalimumab. These alleles may encode peptide-binding grooves that limit the presentation of anti-drug epitopes to T cells, thereby reducing immunogenicity and enhancing drug efficacy. In contrast, the HLA-DRB1*03 and HLA-DRB1*011 alleles are considered detrimental factors that reduce drug efficacy. In anti-infliximab and anti-adalimumab antibody-positive patients, the high frequency of HLA-DRB1*03 was further correlated with poor treatment responses, thus indicating its critical role in modulating immunogenicity and hindering the effectiveness of therapy (54). Another genetic variant, HLA-DQA1*05, has been identified as having significantly increased immunogenicity, which reduces the efficacy of biological therapies (55). According to previous research findings, the frequency of the HLA-E*01:01/01:01 genotype was significantly lower in patients with RA than in the control group. Patients with the HLA-E*01:01/01:01 genotype typically exhibit a better European league against rheumatism response (EULAR) of anti-TNF-α therapy (56). In contrast, carriers of the HLA-E*01:03 allele generally show a poor response to anti-TNF treatment. The HLA-E*01:03/01:03 genotype was also overrepresented among non-responders compared to HLA-E*01:01/01:01 homozygotes. Additionally, patients carrying the HLA-E*01:03:01/01:03:01 genotype frequently demonstrate better responses than those carrying other genotypes (56). The potential mechanisms by which *HLA-E* polymorphisms influence anti-TNF-α efficacy may involve differential immune regulation mediated by specific alleles. HLA-E*01:01, associated with lower cell surface expression of protein, reduces inhibitory signals to NK and T cells by limiting binding to the inhibitory receptor CD94/NKG2A, thereby enhancing immune regulation and promoting autoreactive T cell clearance. In contrast, HLA-E*01:03, characterized by higher expression, strengthens inhibitory signals through CD94/NKG2A, impairing NK and T cell function and compromising immune responses. Additionally, HLA-E*01:01 may more effectively activate regulatory CD8+ T cells (Tregs), enhancing their suppression of pro-inflammatory CD4+ T cells, while HLA-E*01:03 weakens this suppressive function, leading to increased inflammation. Furthermore, HLA-E*01:01 promotes an anti-inflammatory cytokine environment by upregulating cytokines such as IL-10 and downregulating pro-inflammatory cytokines, including TNF-α and IL-6, whereas HLA-E*01:03 elevates pro-inflammatory cytokine levels, exacerbating immune dysregulation. HLA-E*01:01 may also enhance immune regulation through more efficient peptide presentation, while HLA-E*01:03 exhibits reduced antigen presentation efficiency. Further studies are needed to fully elucidate these mechanisms and their impact on anti-TNF-α therapy (56). In a conclusion, these findings therefore emphasize that genetic screening for HLA alleles can predict a patient’s response to adalimumab and other similar biological agents, enabling clinicians to select more effective therapeutic options.

Some non *HLA* genes are also associated with treatment response in RA. Research indicates that the natural killer group 2 member D (*NKG2D*) gene variants (rs2255336 GG and rs1049174 CC genotypes) are associated with diminished responses to anti-TNF-α agents. The G allele of rs2255336 and C allele of rs1049174 have been linked to poor therapeutic outcomes, as noted by the European League Against Rheumatism (EULAR). Interestingly, heterozygous genotypes tended to yield better clinical efficacy than homozygous genotypes, reinforcing the concept that genetic diversity may positively influence drug responsiveness (53). *NKG2D*, which is expressed on NK and T cells, interacts with *MICA* to modulate NK cell activity and T cell responses (71), promoting inflammation through cytokine release (33, 57). Overexpression of the NKG2D ligand sMICA in the RA synovial fluid suggests its important role in maintaining an inflammatory state (72). SNPs in *MICA* (rs1051792) also have an influence on the efficacy of anti-TNF-α therapy. Increased *MICA* expression is often associated with poor clinical response to these therapies, with heterozygous genotypes demonstrating better treatment responses than their homozygous counterparts. This suggests that genetic heterozygosity may provide a protective benefit by modulating the immune signaling pathways in response to TNF-α inhibition (33, 57). PDZ domain containing 2(*PDZD2*) SNPs (rs1532269) have been implicated in reduced efficacy of anti-TNF-α therapy, likely due to their role in regulating metabolic pathways such as insulin secretion. Early RA is often characterized by inflammation-driven insulin resistance, and excessive insulin levels may indirectly attenuate the therapeutic effects of anti-TNF-α agents (58). In contrast, variants in the eyes absent homolog 4(*EYA4*)gene (rs17301249) are associated with an improved response to anti-TNF-α therapy. *EYA4* encodes a co-transcription factor that enhances the production of interferons and C-X-C motif chemokine ligand 10 (CXCL10), both of which are involved in the modulation of inflammatory responses. This suggests that patients with beneficial *EYA4* SNPs may experience amplified therapeutic effects because of their enhanced ability to regulate cytokine production during treatment (58). The *IL17A-152* G/A polymorphism is associated with enhanced response to both biological agents and methotrexate (MTX) treatment. Similarly, the *IL17F 7383* A/G polymorphism has been linked to an improved response to biological therapy. In contrast, the *IL17F 7488* A/G polymorphism significantly reduces the likelihood of a favorable response to biological treatment while enhancing the response to MTX (59). Additionally, past research has shown that genetic variations in NLR family pyrin domain containing 3(*NLRP3*)are related to the susceptibility to RA and response to anti-TNF therapy. These genetic variations may influence gene expression and treatment outcomes in patients (60).

Importantly, variability in gene-associated drug efficacy underscores the value of integrating genetic testing into clinical practice. The identification of genetic predictors, such as *HLA* alleles, *MICA* and *NKG2D* SNPs, or other immune-related gene variants, may facilitate the stratification of patients with RA into subgroups based on their likelihood of responding to specific therapies. For instance, patients with poor-response genotypes may be guided toward alternative treatments such as JAK inhibitors or IL-6 receptor antagonists. Conversely, patients harboring favorable genotypes can be prioritized for anti-TNF-α therapies, ensuring efficient resource allocation and maximizing clinical benefit. These genetic insights provide avenues for developing next-generation therapies designed to overcome immunogenicity and resistance.

## Future direction

RA is a highly heterogeneous disease (73, 74). Emerging technologies, including single-cell multi-omics and CRISPR-based functional genomics are poised to redefine our understanding of RA pathogenesis and accelerate targeted therapy development. Single-cell RNA sequencing (scRNA-seq) and spatial transcriptomics have uncovered unprecedented cellular diversity in RA synovium, peripheral blood, and synovial fluid. For instance, the identification of 18 transcriptionally distinct cell clusters in RA synovium provides a roadmap for dissecting disease mechanisms and identifying cell-specific therapeutic targets. ACPA+ and ACPA- RA subtypes exhibit divergent B-cell differentiation trajectories and synovial immune landscapes, with HLA-DRB5+ memory B-cell depletion in ACPA- patients implicating a genetic basis for immune dysregulation (27, 75). Spatial transcriptomics further resolves niche-specific interactions, including age-associated B cells (ABCs) co-localizing with pro-inflammatory fibroblasts to amplify osteoclastogenesis (76). Future integration of single-cell epigenomics and proteomics will delineate regulatory networks driving pathogenic cell states, while AI-driven analysis of multi-omics datasets could predict patient-specific therapeutic vulnerabilities.

CRISPR-Cas9 technology enables systematic dissection of RA-associated genetic variants and their mechanistic contributions. For example, CRISPR-mediated knockout of miR-155 suppresses macrophage-driven inflammation (77). Future studies could explore the potential of editing *PTPN2/22* to modulate T-cell activation thresholds, a hypothesis derived from their known roles in autoimmune signaling pathways. Similarly, correcting MYC/FOXO1 chromatin aberrations via CRISPR-interference might restore immune homeostasis in CD4+ T cells, though experimental validation is needed to confirm this mechanism (78). Targeting “shared epitope” HLA alleles or non-HLA loci could mitigate genetic risk in predisposed individuals. However, challenges remain in translating CRISPR-based strategies to clinical applications, including delivery efficiency, off-target effects, and heterogeneity in patient genetic architectures. Combinatorial approaches, including coupling CRISPR screens with single-cell functional genomics, may identify master regulators of pathogenic pathways across RA subtypes.

The convergence of single-cell multi-omics, spatial mapping, and CRISPR validation platforms will enable molecular reclassification of RA into mechanistically defined endotypes. For example, synovial enrichment of IL1Bhi macrophages and exhausted CD8+ T cells (Texs) in PD-1 inhibitor-associated arthritis (PD-1-IA) suggests distinct therapeutic requirements compared to classical RA (79). Future studies should prioritize longitudinal multi-omics profiling of large patient cohorts to capture dynamic shifts in cellular ecosystems during disease progression and therapy. Clinically, these advances will drive the development of biomarker-guided therapies, including biologics or CRISPR-engineered cell therapies, tailored to individual molecular signatures (76).The integration of spatial transcriptomics, single-cell epigenomics, and high-throughput CRISPR screening will generate 4D maps of RA synovium, linking genetic variants to spatially resolved cellular interactions. However, scalability, computational integration of multi-modal datasets, and ethical considerations in gene editing remain critical hurdles.

## Discussion

**Figure 1:**
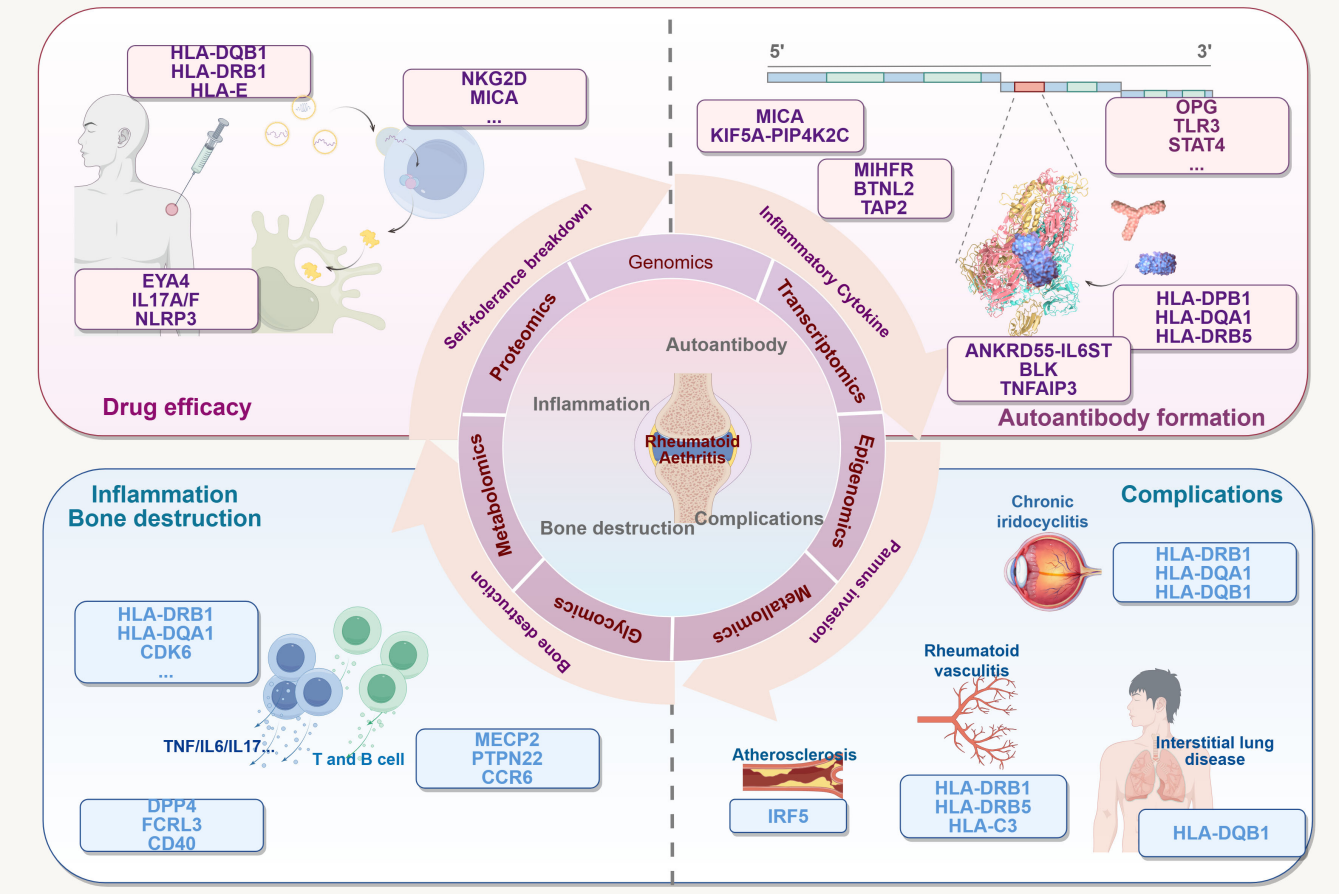
Multiple connections between gene variants and RA. The existence of variants in many genes may be related to several processes of RA: (1) it enhances susceptibility to RA and the process of autotolerance breaking. (2) It promotes antigen presentation and formation of autoimmune T and B cells. The potential consequences may include the formation of a variety of autoantibodies, activation of inflammatory cells and pro-inflammatory factors, mediation of chronic inflammation, and destruction of bones and joints. (3) It affects the efficacy of multidrug therapy for the treatment of RA. It affects multiple tissue diseases associated with RA (4). This Figure made by Figdraw.

The genetic landscape of RA is complex and involves a myriad of gene variants that influence the disease through various pathways, including autoantibody formation, self-tolerance breakdown, inflammation, and bone destruction. Although many gene variants have been directly linked to RA, it is important to consider that several genes, although not directly associated with RA, play well-documented roles in other inflammatory and autoimmune diseases (see **Figure 1**). These genes may offer insights into RA pathogenesis via shared inflammatory pathways, thereby broadening our understanding of RA and its underlying mechanisms. Also, we have made every effort to maintain the currency of gene research. For some interesting genes, we look forward to the publication of valuable updates in the future. (see **Table 1**). For instance, the release of high-mobility group box-1 (HMGB1) from the nucleus into the cytoplasm upon nuclear injury indicates a potential inflammatory mechanism in RA. When HMGB1 binds to advanced glycation end-product-specific receptors (AGER) on macrophages, it activates the stimulator of interferon genes 1 pathway, driving the release of pro-inflammatory cytokines such as TNF-α and IL-6, thus promoting inflammation (77). Similarly, the mismatch repair protein MSH6, in partnership with MSH2, participates in repairing oxidative stress-induced DNA damage and regulates transcription levels, potentially affecting cellular inflammation and epigenetic processes (80). Another example is the CCL2 promoter, which contains an A/G polymorphism that enhances binding of the paired related homeobox 1/Pre-B-cell leukemia homeobox transcription factor to TALE homeodomain protein-binding sites, thereby regulating CCL2 production and promoting inflammation (81, 82). These examples highlight the indirect yet significant role that such gene variants may play in RA, emphasizing the need to further explore their contributions.

Moreover, although significant advancements have been made in regard to understanding the genetic contributions to RA, translating these findings into effective therapeutic predictions remains a challenge. Genetic susceptibility to RA often results from multiple gene mutations, each contributing only modestly to the overall disease risk. These cumulative effects underscore the importance of considering polygenic interactions and environmental influences when studying genetic susceptibility. The role of rare gene variants in larger populations represents a promising avenue for future research to identify novel therapeutic targets. However, it is crucial to recognize that the impact of single gene mutations is limited. Thorough investigations into rare variants using comprehensive approaches such as whole-genome sequencing and bioinformatics could yield valuable insights.

Furthermore, epigenetic mechanisms that encompass modifications beyond DNA sequence changes offer another layer of understanding. These mechanisms clarify the influence of genetic factors on RA. It is vital to consider the cellular and tissue heterogeneity inherent in genetic studies, as selecting appropriate cell types can dramatically impact the interpretation of the genetic and biological mechanisms underlying RA. Integrating genetic analyses with insights from epigenetics, advanced sequencing technologies, and systems biology could enhance our understanding of RA. This multidisciplinary approach may pave the way for more precise and effective interventions that align with the growing emphasis on precision medicine. Predicting genetic mutations and their impacts allows for preemptive disease interventions, thus offering a pathway for personalized treatment strategies.

In conclusion, we focused on over 40 genes rigorously selected based on high-quality literature evidence, which are closely associated with genetic susceptibility to RA, inflammation and bone destruction, autoantibody production, disease complications, or clinical therapeutic responses. These genes were prioritized through a systematic review of human and experimental studies, aligning with our team’s expertise in RA genetics and preliminary research findings. This curated selection not only consolidates robust genetic associations but also provides a theoretical foundation for future clinical research and the development of precision therapeutics. Furthermore, these genes serve as critical candidates for elucidating RA heterogeneity and advancing mechanistic studies, thereby supporting our ongoing efforts to bridge genetic discoveries with personalized diagnostic and therapeutic strategies. Understanding the biological basis of genetic variation in RA not only aids in disease prevention but also supports the development of tailored therapeutic interventions, marking a significant step towards achieving the goals of precision medicine.
